# Fluoxetine ameliorates dysbiosis in a depression model induced by chronic unpredicted mild stress in mice

**DOI:** 10.7150/ijms.37322

**Published:** 2019-09-07

**Authors:** Lijuan Sun, Haohao Zhang, Ying Cao, Chenchen Wang, Changhai Zhao, Huaning Wang, Guangbin Cui, Meixia Wang, Yan Pan, Yupeng Shi, Yongzhan Nie

**Affiliations:** 1 State Key Laboratory of Cancer Biology, National Clinical Research Center for Digestive Diseases and Xijing Hospital of Digestive Diseases, The Fourth Military Medical University, Xi'an, China; 2 Department of Clinical Nutrition, Xijing Hospital, The Fourth Military Medical University, Xi׳an, China; 3 Department of Psychiatry, Xijing Hospital, The Fourth Military Medical University, Xi'an, China; 4 Department of Radiology, Tangdu Hospital, The Fourth Military Medical University, Xi'an, China

**Keywords:** fluoxetine, depression, gut-brain axis, stress, microbiota

## Abstract

**Background**: Accumulating evidence has shown that neuropsychiatric disorders are associated with gut microbiota through the gut-brain axis. However, the effects of antidepressant treatment on gut microbiota are rarely studied. Here, we investigated whether stress led to gut microbiota changes and whether fluoxetine plays a role in microbiota alteration.

**Methods**: We investigated changes in gut microbiota in a depression model induced by chronic unpredicted mild stress (CUMS) and a restoration model by applying the classic antidepressant drug fluoxetine.

**Results**: We found that stress led to low bacterial diversity, simpler bacterial network, and increased abundance of pathogens, such as *Escherichia/Shigella*, and conditional pathogens, such as *Enterococcus*, *Vagococcus*, and *Aerococcus.* However, these changes were attenuated by fluoxetine directly and indirectly. Furthermore, the correlation analysis indicated strong correlations between gut microbiota and anxiety- and depression-like behaviors.

**Conclusions**: This study revealed that fluoxetine led to restoration of dysbiosis induced by stress stimulation, which may imply a possible pathway through which one CNS target drug plays its role in reshaping the gut microbiota.

## Introduction

Recently, documented evidence has shown that the bidirectional interaction between the gut and brain is associated with the maintenance of host nervous system health 1. The gut-brain axis plays a critical role in orchestrating brain development, behaviors, diseases, and signals from the central nervous system (CNS) have also been shown to influence gastrointestinal physiology, motility, and diseases, which in turn regulate CNS function 2. Intestinal microbes are emerging as an important regulator of these interactions 3. Gut microbiota is implicated in the manifestation and etiopathogenesis of neurodegenerative and neural diseases, such as Parkinson's disease 4, Alzheimer's disease 5, autism 6 and depression 7. Interestingly, several nervous disorders also lead to dysbiosis of gut microbiota, including Parkinson's disease 8, multiple sclerosis 9, autism 10, anxiety, and depression 11.

Over the last three decades, considerable progress has been made in understanding how microbiota-induced gut signals are integrated by the CNS. Evidence in animals and humans has indicated that gastrointestinal factors may interact with the brain via the vagus nerve 12-14, neurotransmitters 15, hormones, neuropeptides 16, immune signaling and microglia activation from microbiota 13,14,17. The pathway through which nervous disease leads to dysbiosis is not well understood. It is generally recognized that CNS exerts its effects via the hypothalamic-pituitary-adrenal (HPA) axis and sympathetic branch of the autonomic nervous system (SNS) 18.

Depression is one of the most common psychiatric disorders, and its prevalence ranges from 7-12% in men and 20-25% in women 19. It represents a large health and economic burden; however, few novel therapeutics have been developed due to limited understanding of the pathophysiology of depression. Recent studies have identified the role of microbiota in depression. Compared to conventionally colonized controls, germ-free (GF) mice exhibit substantial alterations in behaviors and neuropathologies that are relevant to psychiatric disorders 20. In addition, intestinal colonization with probiotics produces anti-depressive effects in response to stress 12. Humans with major depressive disorders (MDD) harbor microbiota with reduced diversity, distinct from that of healthy subjects 11. Moreover, fecal microbiota transplantation in GF mice with microbiota derived from depression patients resulted in depression-like behaviors 21. These results collectively support the hypothesis that gut bacteria influence responses to physical and psychological stress. Nevertheless, it remains debatable if alteration in gut microbiota is an initial or subsequent factor in nervous disorders.

Chronic unpredicted mild stress (CUMS) is a widely accepted approach in inducing depression-like behaviors in rodents 22. Fluoxetine, the most widely used antidepressant, increases serotonergic neurotransmission through selective inhibition of neuronal reuptake of serotonin. In the present study, we investigated changes in gut microbiota in a depression model induced by chronic unpredicted mild stress (CUMS) and a restoration model treated with a classic antidepressant drug fluoxetine, and we found that stress led to dysbiosis in gut microbiota and fluoxetine ameliorated the alteration.

## Materials and Methods

### Animals

Male adult C57/6 mice (8 weeks old) were used in all experiments. The mice were housed under a constant temperature of 24 °C with a 12:12 h dark:light cycle. All mice were provided with regular chow and water *ad libitum*, except during food or water deprivation stress. All mice were fed a diet that was mainly composed of corn, soybeans, bran and fishmeal, supplemented with multivitamins, bone powder and trace elements. The diet contained approximately 10% total calories from fat, 20% from protein and 70% from carbohydrates.Weight measurement: After one week of adaptation, the body weight of each mouse was weighed at 8:00 am on the same day every week during the 5 weeks of intervention. When being weighed, the mouse was captured and placed in a box, and then the value was read and recorded.

### Experimental group

Thirty mice were randomly assigned to three different groups: non-stress (n = 10; Control+PBS, i.g.), CUMS + vehicle (n = 10; CUMS+PBS, i.g.), and CUMS + fluoxetine treatment [n = 10; CUMS+12 mg/kg fluoxetine (Merck, USA), i.g.]. For the three groups, PBS or PBS+fluoxetine were intragastrically administered 1 hour before the CUMS procedure over the courses of 3 weeks. The dose volume for gavage administration was adjusted to 0.5ml. Fluoxetine was first dissolved in DMSO and PBS was added to 0.5 ml.

### Chronic unpredictable mild stress model of depression

**Figure 1:**
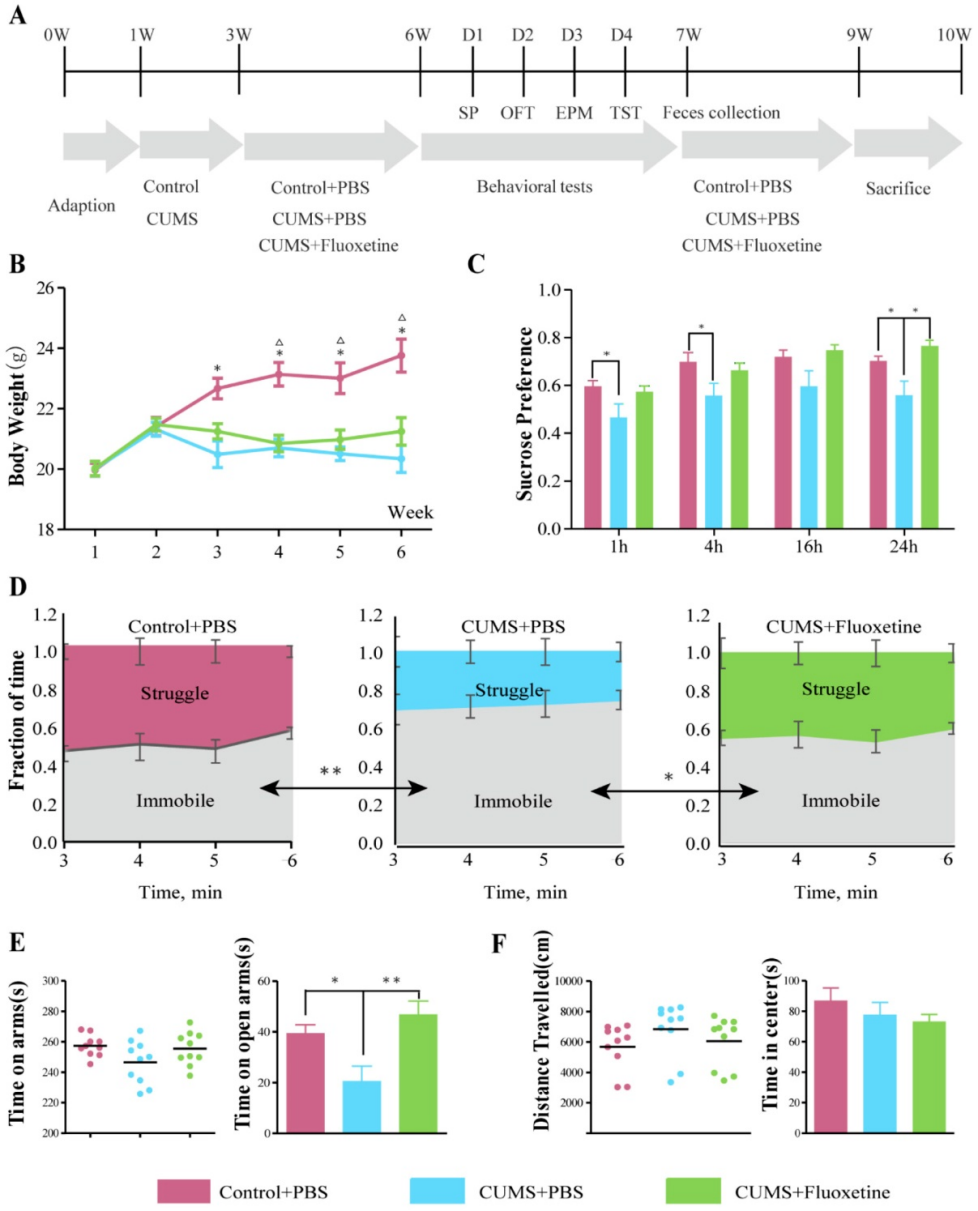
** Fluoxetine showed a significant antidepressant and mild anti-anxiety effects in CUMS mice.** (A) Experimental design of the study. (B) Body weight was measured weekly in all groups during the 6 weeks of CUMS.*P < 0.05, control+PBS*vs.* CUMS+PBS group; ^△^*P*<0.05, control+PBS*vs.*CUMS+fluoxetine, as measured by independent samples *t*-test. (C) SP at 1 h (*F*_2,28_=3.340, *P*=0.051), 4 h (*F*_2,28_=3.252, *P*=0.054), 16 h (*F*_2,28_=2.645, *P*=0.089), and 24 h (*F*_2,28_=4.928,* P*=0.015). (D) Time spent struggling and immobile in the TST (*F*_2,28_=8.572,* P*=0.001). (E) Time spent in all arms (*F*_2,28_=2.650,* P*=0.089) and in open arm in the EPM (*F*_2,28_=10.866,* P<*0.001). (F) Distance travelled (*F*_2,28_=1.299,* P*=0.289) and time spent in center in OFT (*F*_2,28_=0.768,* P*=0.523). Data are presented as mean ± S.E.M. Differences between the three groups were measured by one-way ANOVA. Time spent in arms and distance travelled are covariates for time spent in open arms and time in center respectively. *P < 0.05 **P < 0.01, between two groups, as measured by independent samples *t*-test. CUMS: chronic unpredictable mild stress; EPM: elevated plus maze; OFT: open-field test; SP: sucrose preference; TST: tail-suspension test.

The CUMS model was applied to mice for 6 weeks, using a protocol that has been reported previously 22,23. Briefly, mice were subjected to the mild stress protocol in an unpredictable manner for 5 weeks. The protocol consisted of seven stressors: restraint stress for 5 h, overnight illumination for 8 h, horizontal oscillation for 20 minutes, cage tilting at 45° for 24 h, soiled cage for 24 h, food deprivation for 24 h, and water deprivation for 24 hr. The vehicle or fluoxetine treatment was administered *via* intragastric gavage (i.g.) from week 3-6 following stress exposure. Figure 1A shows the experimental design for this study.

### Behavioral tests

#### Tail Suspension Test

Tail Suspension Test was designed and widely used to test the depression-like behaviors in mice. The mice were suspended 50 cm above ground by adhesive tape that was attached approximately 1 cm from the base of the tail for 6 min. The behavior of each mouse was video-recorded, and the results were scored by an experimenter blind to the groups using the time-sampling technique. The first 2 minutes of the test were considered as habituation. Total IT in the final 4 min of the test was recorded. Immobility was defined as no movement and regarded as a depression-like behavior 24.

#### Sucrose Preference

The two-bottle choice for assessing sucrose preference is another popular test to investigate changes in positive affective stimuli in rodents. We used the test to evaluate the depression-like behaviors in mice. Mice were individually housed in double-grommet ventilated Plexiglas cages to monitor individual fluid consumption. Prior to testing, mice were deprived of water and food for 12 h, followed by 1% sucrose solution for 12 h for habituation. Following this, mice were housed individually and given a free choice between two bottles (150 ml 1% sucrose solution or 150 ml tap water). The position of each bottle was exchanged after 6 h to avoid any side-preference effects. SP was calculated as SP (%) = sucrose intake (g)/(sucrose intake (g) + water intake (g)) × 100% 25.

#### Elevated Plus Maze

In a depression model, experimental animals are often accompanied by an increase in anxiety-like behavior. We also used behavioral experiments to detect anxiety-like behaviors. The elevated plus maze is widely used to assess behaviors in rodents and has been validated to assess the anti-anxiety effects of drugs. The test consists of an elevated, plus-shaped apparatus with two open and two enclosed arms. Mice were placed in the central platform with their nose facing a closed arm. Behavior was recorded for 5 min by an overhead color CCD camera. All mice were tested once between 12:00-16:00. Time spent in open and closed arms, and entries into open and closed arms were calculated. The total time spent in arms was used as a measure of general activity. Time spent in open arms (OAT) was used as an index of anxiety-like behavior 26.

#### Open Field Test

It is usually used to observe autonomous behavior, inquiry behavior and tension of experimental animals in new environments. We also used this test to evaluate the anxiety-like behaviors in mice. Square locomotor boxes from Med Associates (L 27.3 × W27.3 × H 20.3 cm, St. Albans, VT, United States) were used to monitor locomotor activity. All animals were moved to the testing room for 24 h prior to testing to avoid the measurement of locomotor activity associated with novelty and/or anxiety. During the test session, time spent in the center of the area was recorded for 15 min. All testing was conducted during the dark/active phase 27.

### Fecal collection, DNA extraction, PCR amplification, and 16S sequencing

All fecal samples were collected fresh, and then stored at -80 °C. Next, DNA extraction was performed according to Godon et al. 28. All samples were sequenced using the IlluminaMiseq platform according to the manufacturer's instructions.

### Bioinformatics and Statistical Analysis of 16S sequencing data

Quality control of the raw sequencing data was performed as described by Zhang 29. Quality-filtered sequences were clustered into unique sequences and sorted in order of decreasing abundance to identify representative sequences using UPARSE according to the UPARSE OTU analysis pipeline. Singletons were omitted in this step. OTUs were classified based on 97% similarity after chimeric sequences were removed using UPARSE. The phylogenetic affiliation of each 16S rRNA gene sequence was analyzed by the RDP Classifier against the RDP database (RDP Release 11) using a 70%confidence threshold.

Sample diversity was assessed on the basis of the nonparametric Shannon-Wiener diversity index, which was calculated using QIIME. The QIIME pipeline was also used to generate PCoA plots to visualize the un-weighted UniFrac dissimilarity. LEfSe was used to detect taxa with differential abundance among groups. All bar and PCoA plots were generated in R.

### Co-occurrence network analysis method

Bacterial genus occurring in more than half of the samples was used for network analysis. Non-random co-occurrence patterns of the selected genus were tested with the checkerboard score (C-score) under a null model. Spearman's rank correlations between the selected genus were calculated. A valid co-occurrence event was considered to be a robust correlation if the Spearman's correlation coefficient was *Spearman's >0.7 or <- 0.7* with a significance of *P*< 0.05. Correlation networks were constructed with the robust correlations as weighted edges using Gephi software. Ten thousand Erdös-Réyni random networks with the same number of nodes and edges as the empirical networks were generated using the R package igraph.

### Statistical analysis

All statistical analyses were performed using SPSS version 19.0 (IBM, Armonk, NY, USA). The data were expressed as the mean ± standard error of the mean (S.E.M.). Differences among three groups were assessed using one-way ANOVA. Differences between two groups were assessed using independent-sample *t*-tests. Differences were considered statistically significant when *P*<0.05.

## Results

### Fluoxetine showed significant antidepressant and mild anti-anxiety effects in CUMS mice

Depression- and anxiety-like behaviors were examined by four behavioral tests, i.e., sucrose preference test (SPT), tail suspension (TST), elevated plus maze (EPM) and open field test (OFT) following the protocol shown in Figure 1A. The mean body weight in the CUMS+phosphate-buffered saline (PBS) and CUMS+fluoxetine groups was significantly lower than that in the control group (Figure 1B). Sucrose preference (SP) was significantly decreased at 1 h (*t*=2.129, *P*=0.047), 4 h (*t*=2.204, *P*=0.041), and 24 h (*t*=2.373, *P*=0.029) after being given a free choice between two bottles in the CUMS+PBS group compared to the Control+PBS group, but fluoxetine ameliorated the reduction in sucrose preference at 24 h (*t*=2.259, *P*=0.037) (Figure 1C). Furthermore, immobility time (IT) increased after CUMS (*t*=-3.506, *P*=0.003) and fluoxetine significantly reduced IT (*t*=-2.308, *P*=0.033) in the TST (Figure 1D). Time spent in the open arms (OAT) was significantly decreased following CUMS in EPM (*t*=-2.693, *P*=0.015), and this effect was ameliorated following fluoxetine treatment (*t*=4.029, *P*=0.001) (Figure 1E). There was no significant difference in locomotion and time spent in the center (CT) between the three groups, as measured in the OFT. These data indicated that fluoxetine exerted a significant antidepressant effect and a tendency towards an anti-anxiety effect, as was consistent with previous reports 21.

### Fluoxetine ameliorated the altered composition, low bacterial diversity and simple bacterial network induced by CUMS

To evaluate whether stress-induced alterations in depression- and anxiety-like behaviors were associated with changes in gut microbiota, 18 individual feces were collected from mice in the three groups and microbiota profiles were analyzed using bacterial taxa 16S rRNA gene amplicon sequencing.

**Figure 2:**
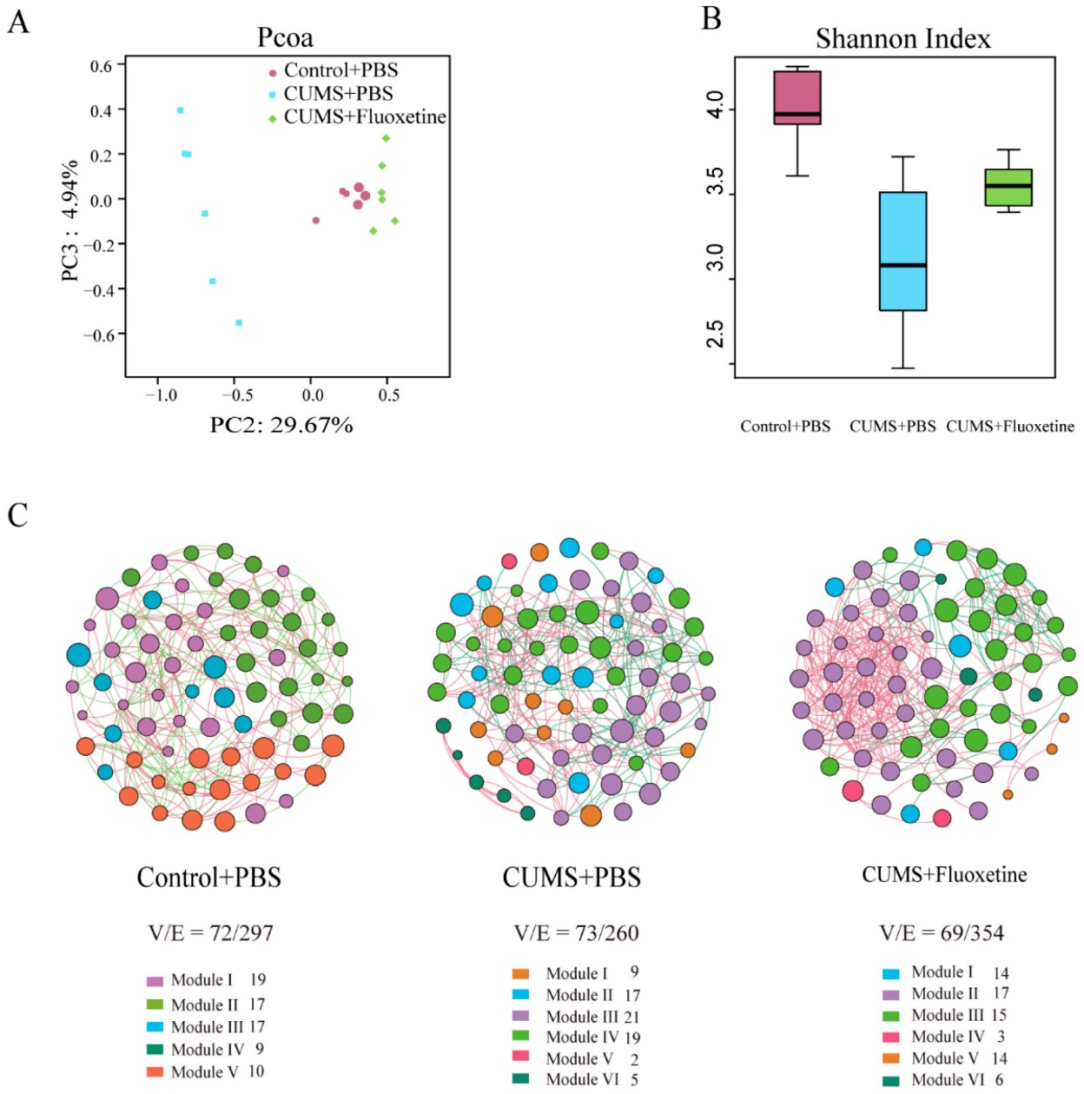
** Fluoxetine ameliorated the altered composition, low bacterial diversity and simple bacterial network induced by CUMS.** (A) Shannon diversity scores. (B) PCoA analysis plots of Bray-Curtis dissimilarity between groups. (C) Network analysis at the genus level. Networks are randomly colored by modules. V: number of nodes. E: number of edges.

The Principle Coordinates Analysis (PCoA) plots of Bray-Curtis dissimilarity among the three groups showed that the dots of the CUMS group were separated from the dots of the control group; however, the dots of the CUMS+fluoxetine group were close to the dots of the control group. This suggested that fluoxetine attenuated the CUMS-induced alteration in gut microbiota composition (Figure 2A). Similarly, the Shannon index showed that bacterial diversity was significantly decreased in the CUMS+PBS group; however, fluoxetine treatment ameliorated this effect (Figure 2B). To determine the co-occurrence pattern of microorganisms in the three groups, we constructed three networks at the genus level, which showed that CUMS-induced mice had a simpler property (V/E=73/260) compared with the control group (V/E=72/297), and fluoxetine restored the sophisticated property in the co-occurrence network (V/E=69/354). This indicated that CUMS induced vulnerability to environmental stress in gut microbiota, and fluoxetine restored the complex property (Figure 2C).

### Fluoxetine remodeled stress-induced dysbiosis directly and indirectly

**Figure 3:**
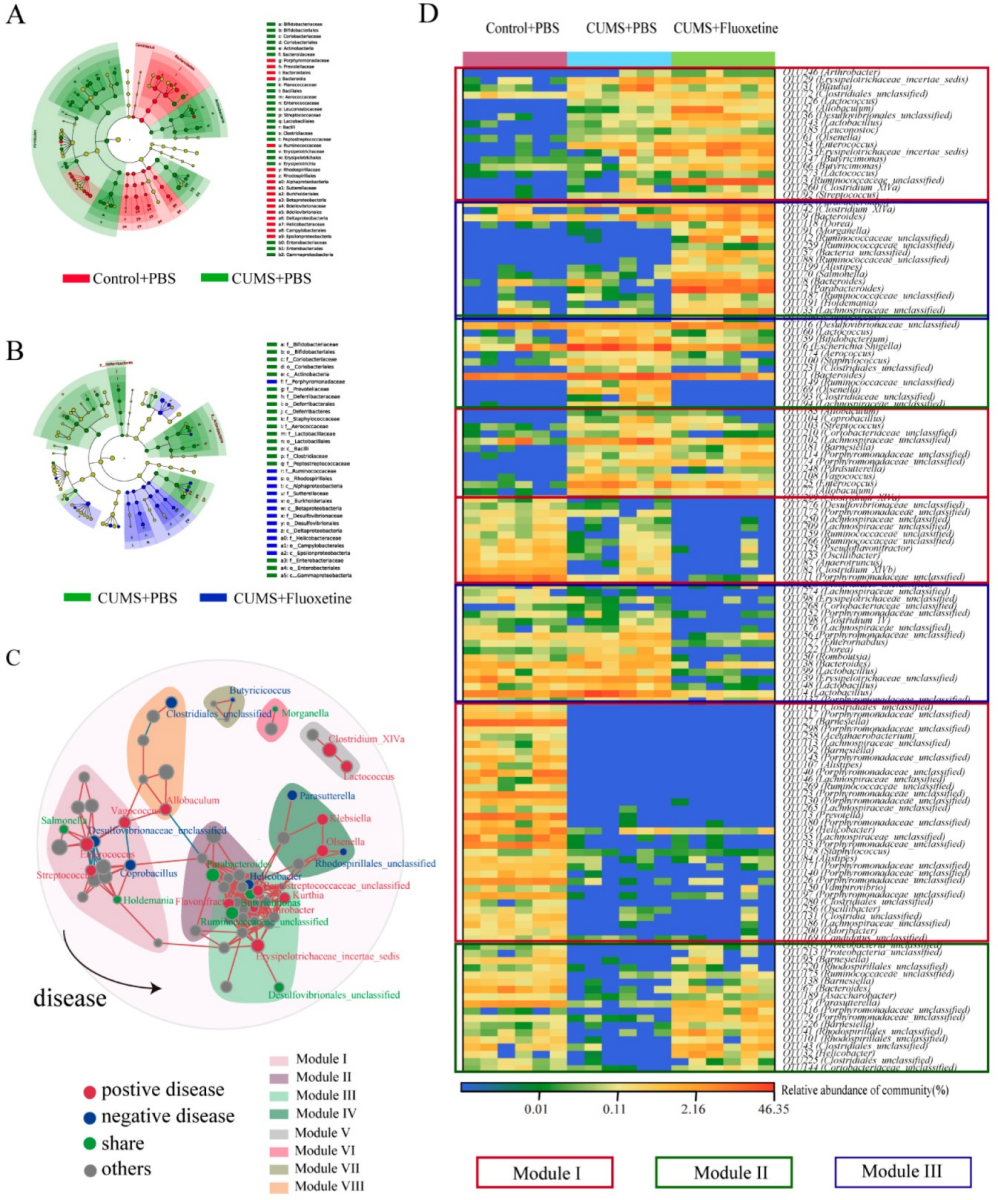
** Fluoxetine remodeled stress-induced dysbiosis (directly and indirectly).** (A) Lefse analysis of microbiomes between control+PBS and CUMS+PBS groups. (B) Lefse analysis of microbiomes and CUMS+PBS and CUMS+fluoxetine groups. (C) The bacterial network associated with depression and fluoxetine. Red dots: bacteria positively associated with depression-like behavior whose abundance was significantly increased in CUMS+PBS mice compared to Control+PBS; Blue dots: bacteria negatively associated with depression-like behavior whose abundance was significantly increased in CUMS+fluoxetine mice; Green dots: bacteria associated with depression and fluoxetine whose abundance was significantly increased in CUMS+PBS mice and decreased in CUMS+fluoxetine mice. Red line: positive correlation; grey line: negative correlation. (D) Heatmap of key OTUs. Red frame: OTUs affected by CUMS but not corrected by fluoxetine. Green frame: OTUs affected by CUMS and corrected by fluoxetine. Blue frame: OTUs not affected by CUMS but influenced by fluoxetine. CUMS: chronic unpredictable mild stress.

The linear discriminant analysis of effect size (Lefse) showed that a higher abundance of bacterial taxon in the CUMS group primarily originated from Firmicutes, Actinobacteria, Erysipelotrichia, and Gammaproteobacteria, whereas a lower abundance originated from Bacteroidetes, Alphaproteobacteria, Betaproteobacteria, Deltaproteobacteria, and Epsilonproteobacteria (Figure 3A). Furthermore, following fluoxetine treatment, dysbiosis was partly corrected. The bacterial taxon rescued by fluoxetine treatment mostly originated from Erysipelotrichia; where part of Proteobacteria was also ameliorated. Alphaproteobacteria, Betaproteobacteria, Deltaproteobacteria, and Epsilonproteobacteria levels were re-elevated after fluoxetine treatment; however, fluoxetine had no effect on Gammaproteobacteria (Figure 3B).

To further observe the modulatory action of fluoxetine on depression-related bacteria, we constructed networks involving all of the bacterial genera in the CUMS+fluoxetine group. We found that a part of depression-related taxon could be directly restored by fluoxetine (green spots). Interestingly, although some depression-related taxa were not directly rescued by fluoxetine (red spots), these bacteria were significantly related to bacteria that were affected by fluoxetine (blue and green spots), which implied that fluoxetine may also impact depression-related bacteria indirectly (Figure 3C).

At the OTU level, we observed some bacteria affected by stress that could not be restored by fluoxetine (red frame), and some bacteria that were altered after stress stimulation but were corrected by fluoxetine (green frame). Additionally, we also observed that some changes were different from the CUMS-induced variation (blue frame), which may be related to the chemical effect of fluoxetine (Figure 3D).

### Fluoxetine recovered depression-specific bacteria at the OTUs level

**Figure 4:**
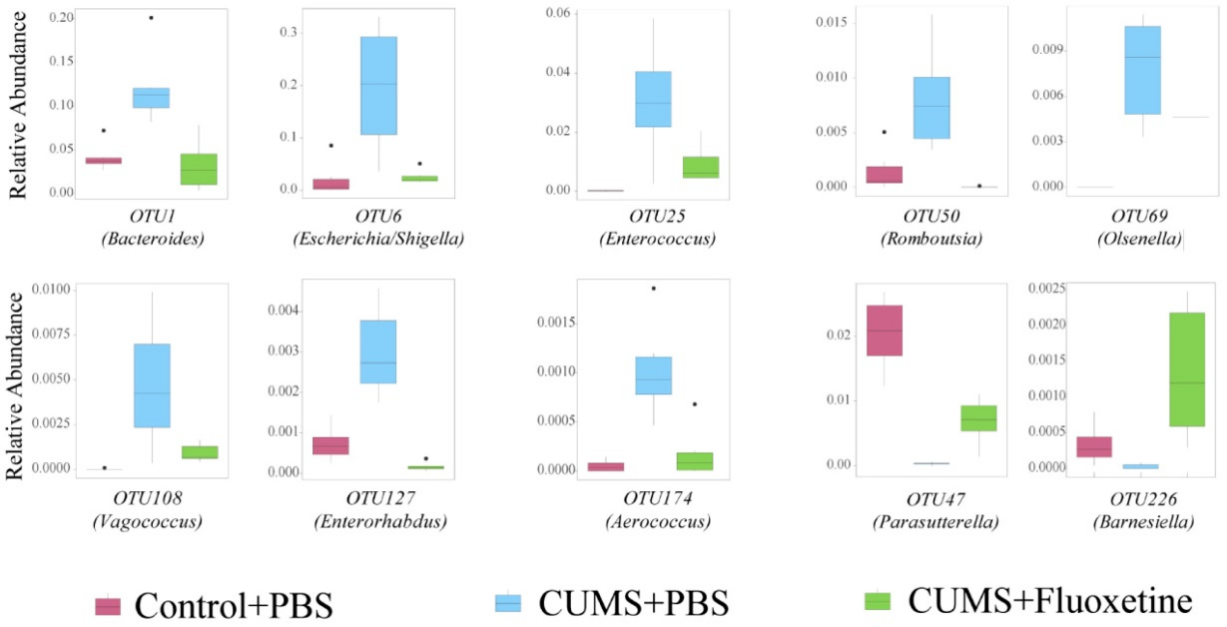
** Fluoxetine recovered depression-specific bacteria at the OTUs level.** Altered composition of gut bacteria with different abundance at the OTUs level.

The microbiota composition between groups was significantly different at the OTU level (Supplementary Figure 1). To further identify the specific bacteria that were rescued by fluoxetine, we selected a bacterial taxon that differed in abundance between the Control+PBS and CUMS+PBS groups, and the CUMS+PBS and CUMS+fluoxetine groups. The abundance of OTU1 (*Bacteroides*), OTU6 (*Escherichia/Shigella*), OTU25 (*Enterococcus*), OTU50 (*Romboutsia*), OTU69 (*Olsenella*), OTU108 (*Vagococcus*), OUT127 (*Enterorhabdus*) and OTU174 (*Aerococcus*) were significantly increased in the CUMS group compared with the control group; however, fluoxetine treatment attenuated this increase. In addition, the abundance of OTU47 (*Parasutterella*) and OTU226 (*Barnesiella*) was decreased following stress stimulation, and fluoxetine significantly ameliorated this reduction (Figure 4). We also observed that some unclassified bacteria could be impacted by CUMS and restored by fluoxetine (Supplementary Figure 2). This indicated that environmental stress may promote the growth of potentially detrimental bacteria and inhibit the growth of potentially probiotic bacteria, and fluoxetine can correct their variation induced by CUMS.

### Depression-specific bacterial genera were linked to anxiety- and depression-like behaviors

**Figure 5:**
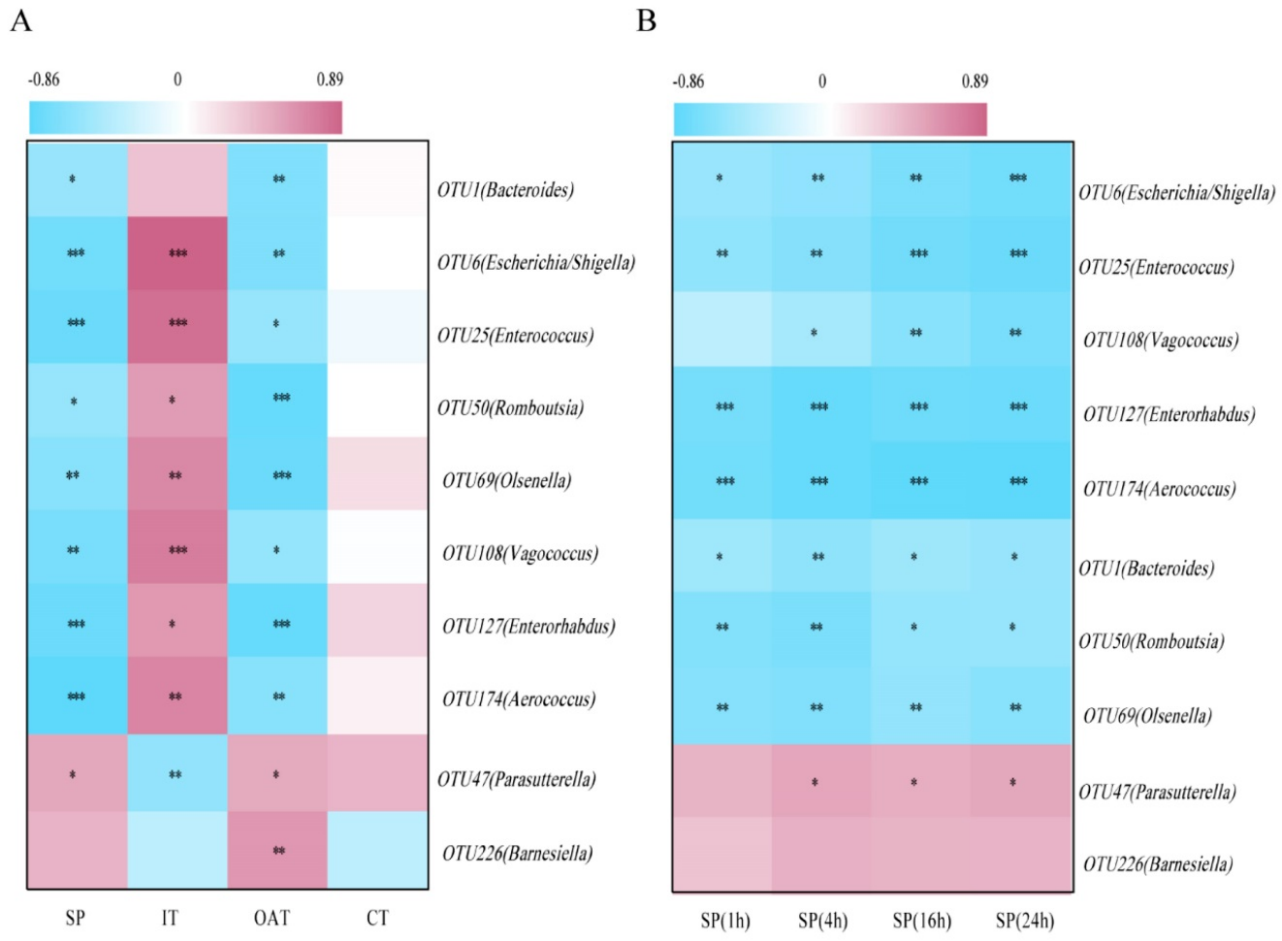
** Depression-specific bacterial genera were linked to anxiety- and depression-like behaviors.** (A) Heatmap of Spearman's rank correlation coefficients between the behavioral indices and bacterial abundance between groups. (B) Correlation analysis between given bacteria and time course of SP. *P < 0.05, **P < 0.01, ***P < 0.001.

To identify correlations between the behavioral parameters and given bacteria, we performed an association analysis. We found that OTU1 (*Bacteroides*), OTU6 (*Escherichia/Shigella*), OTU25 (*Enterococcus*), OTU50 (*Romboutsia*), OTU69 (*Olsenella*), OTU108 (*Vagococcus*), OTU127 (*Enterorhabdus*), and OTU174 (*Aerococcus*) were significantly negatively correlated with SP, OAT in EPM, and CT in OFT, but positively correlated with IT, indicating that these bacteria were potentially detrimental bacteria for anxiety- and depression-like behaviors. More notably, OTU47 (*Parasutterella*) was positively correlated with SP, OAT, and IT, and negatively correlated with IT, indicating that it may be potentially probiotic resistant to stress stimulation (Figure 5A). Correlation analysis between given bacteria and time course of SP was also identified (Figure 5B). OTU6 (*Escherichia/Shigella*), OTU25 (*Enterococcus*) and OTU108 (*Vagococcus*) were more significantly linked to SP over the course of time.

## Discussion

Physiological stress can lead to altered gut microbiota, which can provide feedback and impact gastrointestinal function and stress-induced behavioral states. This study found that long-term stress led to dysbiosis in gut microbiota, whereas fluoxetine rescued the deterioration in microbiota in specific bacteria taxa. It offered a novel perspective through which we can evaluate the application of fluoxetine. This will also uncover a role of gut microbiota in the gut-brain axis.

We found that fecal microbial diversity and the bacterial network were significantly reduced following stress stimulation, which was partially rescued following fluoxetine treatment. Kelly and colleagues 30 reported that depression was associated with a reduced richness and diversity of gut microbiota in rats. However, it has been reported that the Shannon index is unexpectedly higher in patients with MDD 11. At the phylum level, intestinal dysbiosis was characterized by significantly higher abundance of *Firmicutes, Actinobacteria, Erysipelotrichia,* and *Gammaproteobacteria*, and a lower abundance of *Bacteroidetes, Alphaproteobacteria*, *Betaproteobacteria*, *Deltaproteobacteria,* and *Epsilonproteobacteria.* Zheng et al. 21 reported that the abundance of *Actinobacteria* and *Bacteroidetes* increased and decreased respectively in patients with MDD, which is consistent with the results of this study. However, an alteration in the abundance of *Firmicutes* was significantly different from that found in previous studies. In our study, we observed a significant increase in *Firmicutes* abundance in the CUMS group. Jiang et al. 11 reported that *Firmicutes* levels decreased, whereas Zheng et al. 21 found no difference in the overall relative abundance of *Firmicutes* in patients with MDD. Jeffery et al.^31^ reported an increase in *Firmicutes*-associated taxa in a subgroup of patients with irritable bowel syndrome (IBS). Increased *Firmicutes* abundance is associated with metabolic diseases such as obesity 32. Interestingly, obesity and depression show a robust association in epidemiological studies 33. Disparities between the studies may be due to differences in models; however, these findings have consistently supported the hypothesis that stress is linked to alterations in gut microbiota.

We found that CUMS-induced dysbiosis was partially rescued in a specific bacterial taxon by fluoxetine. A recent study has shown that in a depression model of rats, stress increases the abundance of *Deinococcus* and concentration of hexanoic acid, whereas fluoxetine attenuated these alterations, but the role of hexanoic acid in depression is not clear 34. In our study, most of the fluoxetine-induced changes were in the abundance of bacteria from *Erysipelotrichia* and *Proteobacteria* classes*.* At the OTU level, the bacterial taxa in *Bacteroides*, *Escherichia/Shigella*, *Enterococcus*, *Romboutsia*, *Olsenella*, *Vagococcus*, *Enterorhabdus* and *Aerococcus* were significantly increased in the CUMS group compared with the control group, which was attenuated following fluoxetine treatment. *Escherichia/Shigella* are generally harmless, although they can lead to diarrhea, fever, urinary tract infections, and pneumonia. In addition, patients infected by *Shigella* show an increased risk of IBS for 3 years following the initial infection. Interestingly, IBS is a widely accepted gastrointestinal disorder that is accompanied by depression 35. In addition, we detected the overgrowth of some conditional pathogens belonging to three *Firmicutes* phyla, such as *Enterococcus*, V*agococcus,* and *Aerococcus*. Many studies have shown that depression is associated with a chronic low-grade inflammatory response 36, and long-term chronic stress exposure can significantly increase blood-brain barrier permeability 37. This facilitates the translocation of pathogens and their product transfer from the lumen of the intestine to the CNS of the host. We speculate that the relative high abundance of these genera in the CUMS group partially mediates the development of inflammation and may play a pivotal role. More intriguingly, in our study, we found that *Parasutterella* was markedly decreased in the CUMS group and was ameliorated following fluoxetine treatment. Furthermore, its abundance was negatively associated with IT in the TST, suggesting a protective role for this taxon during a stress response. *Parasutterella* is a relatively new genus with few studies assessing its host effects. Zhang et al.[38]have reported that its abundance was decreased in mice with diet-induced obesity. Furthermore, an *in vitro* model showed that *Akkermansia* and *Parasutterella* were accompanied by a slower deconjugation of taurocholic acid 39. These data suggest that *Parasutterella* may have a role in metabolic disorders. These results also provide a list of bacteria that have an effect on depression-like behaviors, and more studies are required to explore the role of these bacteria during a stress response.

In this study, the alteration observed in microbiota could be classified into three subcategories: alteration induced by stress stimulation but not affected by fluoxetine; alteration caused by stress and fluoxetine simultaneously, for which in our study fluoxetine showed a significant effect of restoration. These two subcategories are hugely affected by stress. Stress may also exert its effects *via* the HPA axis and sympathetic branch of the SNS 18. The CNS and gastrointestinal tract are intimately connected, and depression can significantly impact gastrointestinal physiology *via* the actions of norepinephrine and epinephrine. Antidepressant drugs can restore HPA axis function 40 and induce alterations in glucocorticoid hormones. In addition, fluoxetine can affect sympathetic activities, followed by gastric acid secretion and gastric emptying, which can significantly impact gut microbiota 18. Most importantly, this implied that the mechanism of fluoxetine as an antidepressant and selective serotonin reuptake inhibitor may be a possible pathway CNS leads to gut microbiota alteration. The third subcategory is alteration in microbiota not induced by stress but caused by fluoxetine, which is more likely caused by direct chemical action. Non-antibiotic drugs have a significant impact on human gut bacteria, and drugs that target the nervous system inhibit gut bacteria more than other medications 41; however, the effect of fluoxetine on gut bacteria has not been assessed. Fluoxetine has a higher affinity to 5-HT_2A_ and 5-HT_2C_ receptors, but 5-HT_2A_ receptor has been previously described not only in neurons of the CNS but also in cholinergic neurons of intestinal submucosa and muscularis. This may be another mechanism the drug affects gut microbiota. Interestingly, the network analysis showed that this subcategory was significantly associated with the other two subcategories.

There are some limitations of this study. First, referring to the study procedure, undoubtedly, gavage administration of PBS or fluoxetine also induced a stress response, which may be a factor affecting the gut microbiota. An earlier study demonstrated that gavage administration of various vehicles induced a stress response in a volume-dependent fashion 42. Thus, we also treated the mice in the control group with gavage and adjusted the gavage dose to 0.5 ml. Additionally, sucrose administration may be a factor affecting gut microbiota. It has been reported that high sucrose consumption for four weeks could promote gut microbiota dysbiosis, which was characterized as increased *Clostridia* and *Bacilli* and decreased *Lactobacillus* spp. 14. However, we administered to all the mice with sucrose for only 24 hours and collected feces after one week, therefore this has a minor impact on gut microbiota. Second, it is not known whether altered gut microbiota we observed in this study is a causal factor or an accompanying consequence of stress exposure and fluoxetine treatment. We hypothesize that fecal microbiota transplantation may aid with clarification of their relationship. Third, our findings use an animal model; therefore, this may not represent the characteristics of patients with depression. Further clinical studies may be necessary to address this.

In summary, our study investigated changes in gut microbiota in a depression model induced by CUMS and a restoration model by applying the classic antidepressant drug fluoxetine.It demonstrated that fluoxetine led to restoration of dysbiosis induced by stress stimulation. This study contributed to previous reports on the involvement of gut microbiota during stress exposure, and gave us a novel perspective in evaluating the applications of antidepressant drugs on microbiota. Further investigations of the role of altered microbiota with their antidepressant effect are needed to elucidate the accompanied or causal relationships between them, and further studies involving cohorts are also needed.

## Supplementary Material

Supplementary figures.

## Abbreviations

CNS: central nervous system
CUMS: chronic unpredictable mild stress
IBS: irritable bowel syndrome
i.g.: intragastric gavage
Lefse: linear discriminant analysis of effect size
MDD: major depressive disorder
EPM: elevated plus maze
OFT: open field test
OTU: operational taxonomis unit
PBS: phosphate buffered saline
PCoA: principle coordinate analysis
SP: sucrose preference
TST: tail suspension test
