# Supplementary material for: Fluoxetine ameliorates dysbiosis in a depression model induced by chronic unpredicted mild stress in mice
Source: Int J Med Sci. 2019 Sep 7;16(9):1260–70. doi: 10.7150/ijms.37322 (PMC6775263; doi:10.7150/ijms.37322)
Supplement: Supplementary file 1 — Supplementary figures. [file ijmsv16p1260s1.pdf]

Fluoxetine ameliorates dysbiosis in a depression model induced by chronic unpredicted mild stress in mice

Supplementary Figures and Figure Legends

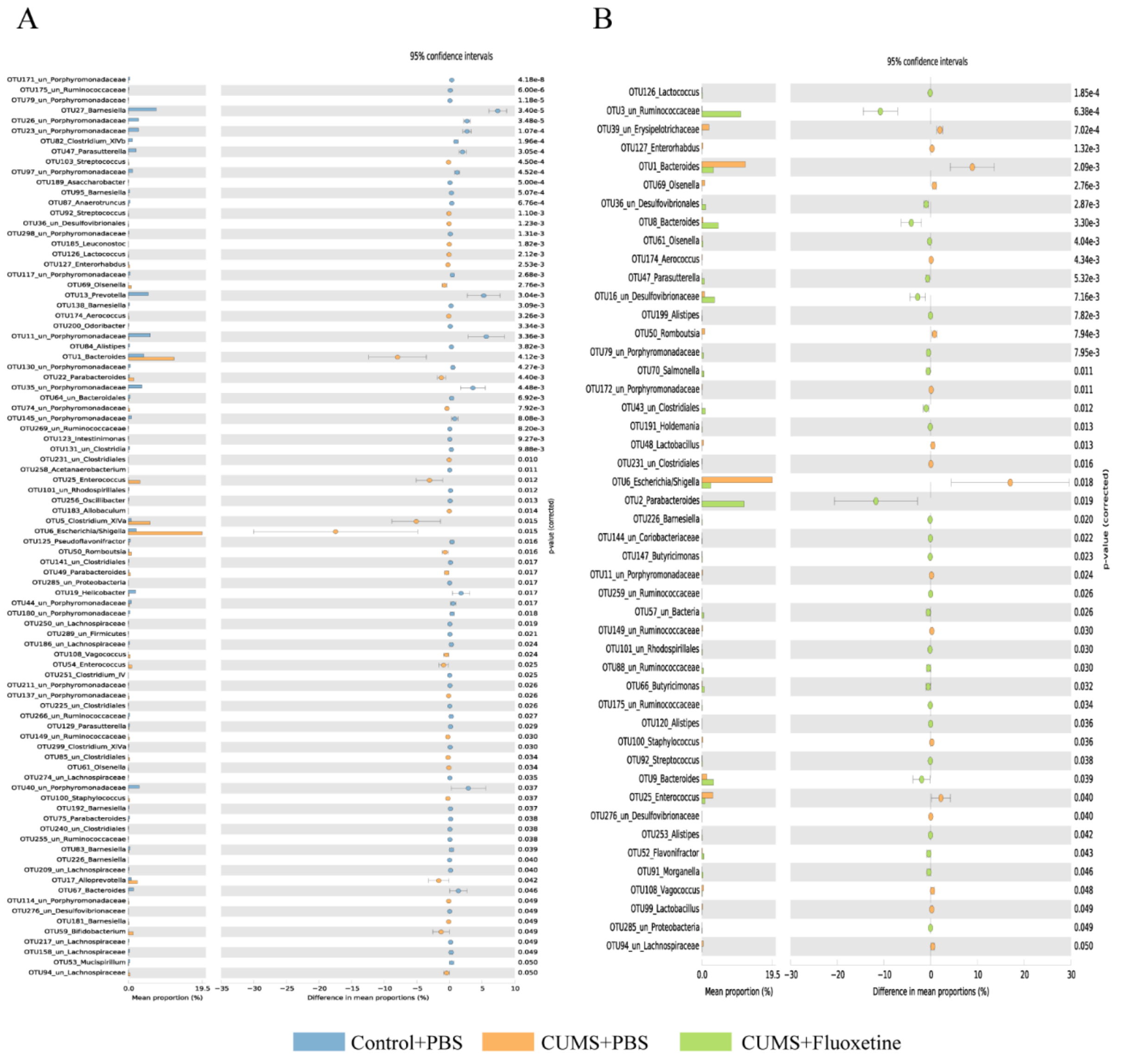

Supplementary Figure 1. OTUs level differences between groups

STAMP software was used to calculate the OTUs proportions. Welch's t-test was used to compare the abundance of bacteria between the Control+PBS and CUMS+PBS groups (A), and CUMS+PBS and CUMS+fluoxetine groups (B). Different genera were assigned to those presenting minimum variation at a significant level of $P < 0.05$. OTU: operational taxonomic unit. CUMS: chronic unpredictable mild stress.

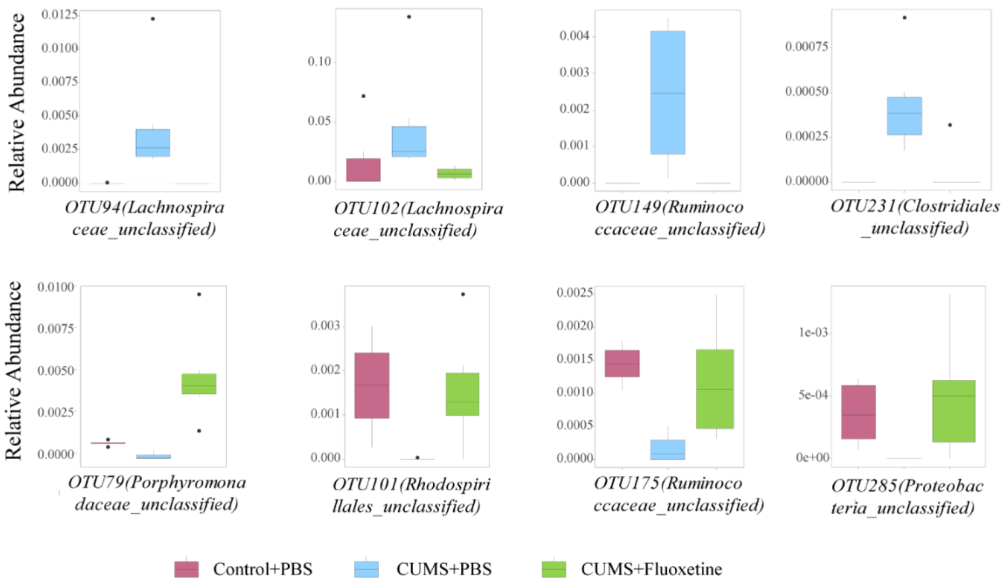

Supplementary Figure 2. Unclassified bacteria affected by CUMS and Fluoxetine

Altered composition of gut bacteria with different abundance at the OTUs level. OTU: operational taxonomic unit.
